# The role of emotion regulation as a mediator between early life stress and posttraumatic stress disorder, depression and anxiety in Syrian refugees

**DOI:** 10.1038/s41398-020-01062-3

**Published:** 2020-11-02

**Authors:** Zaynab Demir, Kerem Böge, Yan Fan, Corinna Hartling, Mazen R. Harb, Eric Hahn, Joachim Seybold, Malek Bajbouj

**Affiliations:** 1 Department of Psychiatry and Psychotherapy, Campus Benjamin Franklin, Charité – Universitätsmedizin Berlin, Corporate Member of Freie Universität Berlin, Humboldt-Universität zu Berlin and Berlin Institute of Health, Berlin, Germany; 2 Charité – Universitätsmedizin Berlin, Corporate Member of Freie Universität Berlin, Humboldt-Universität zu Berlin and Berlin Institute of Health, Berlin, Germany

**Keywords:** Human behaviour, Depression

## Abstract

Early life stress is an important factor in later psychopathology, including symptoms of posttraumatic stress disorder (PTSD), depression, and anxiety. The purpose of the present study was to investigate the effect of early life stress on psychiatric symptoms within a sample of Syrian refugees. In this model, the use of cognitive emotion regulation strategies was assessed as a potential mediator of the relationship between early life stress and current symptoms of PTSD, depression, and anxiety. Bootstrap analyses were generated to test the indirect effect of emotion regulation (Cognitive Emotion Regulation Questionnaire) on the relationship between early life stress (Childhood Trauma Questionnaire), PTSD (Harvard Trauma Questionnaire), depressive (PHQ-9) and anxiety (GAD-7) symptoms in eighty-nine Syrian refugees resided in Germany (*n* = 49) and Jordan (*n* = 40). The indirect effect of maladaptive strategies was significant between early life stress and psychopathology, whereas the mediation effect of adaptive strategies was not significant. The findings provide an evidence that emotional dysregulation is an underlying factor affecting psychological symptoms in refugees with adverse childhood experiences. These results suggest targeting cognitive emotion regulation in prospective prevention and treatment strategies.

## Introduction

There is extensive evidence for interdependencies between stress in early childhood and physical^1^ as well as mental illnesses such as posttraumatic stress disorder (PTSD), depression, generalised anxiety, panic disorder, social phobia, substance use, and personality disorders across the lifespan^2–7^.

Early life stress is the exposure to single or multiple events during childhood that threaten emotional or physical well-being to the extent, that exceeds the child’s coping resources and leads to prolonged phases of stress^8^. Theorists have already observed specificity between certain types of adverse events in childhood and different forms of adulthood psychopathology^3^. For instance, Rose and Abramson^9^ suggested that childhood emotional abuse is more likely to cause cognitive vulnerability to depression than either childhood physical or sexual abuse, because with emotional abuse depressive cognitions are directly provided to the child by the perpetrator. With repeated experiences of childhood emotional abuse, children may begin to make negative reasonings for their occurrence, which then may favor a cognitive style that would cause specific vulnerability to depression^9^. Consistent with these theories, self-criticism was found to be a mediator of the relationship between parental verbal abuse and later depression and anxiety^10^. Other studies lend further support for the mediator role of a negative cognitive style in the link of childhood emotional maltreatment and current depression^11,12^. Even though several potential mediators have been identified in the literature so far, research focusing on cognitive emotion regulation as a mediator of the relationship between early life stress and adult symptom presentations is still scarce.

The term emotion regulation has been used in different ways^13^, but one highly cited definition of emotion regulation is “the ability to respond to the ongoing demands of experience with the range of emotions in a manner that is socially tolerable and sufficiently flexible to permit spontaneous reactions as well as the ability to delay spontaneous reactions as needed”^14,15^. Cognitive emotion regulation or cognitive coping can be defined as the cognitive way of managing the intake of emotionally arousing information^16^. Cognitive processes may help us to regulate the emotions, and not to get overwhelmed by them during or after the experience of threatening or stressful events^17^. A growing body of evidence suggests that by using maladaptive strategies (i.e., self-blame, rumination, catastrophizing, and blaming-others) people may be more vulnerable to psychopathology than others, whereas adaptive cognitive styles (i.e., acceptance, positive refocusing, refocus on planning, positive reappraisal, and putting into perspective) may lead to more resilience to symptoms of psychological distress^18–20^. Previous research showed that trauma, especially enduring or repeated traumatic experiences such as early life stress, seems to compromise the acquisition of appropriate emotion regulation skills^21^. For example, one study showed that sexually abused girls have subsequent difficulties understanding and regulating their emotions compared to nonmaltreated peers^22^. A related study confirms that children who have experienced neglect present less adaptive emotion regulation skills^23^.

Emotional dysregulation originating from early life stress appears to be relevant to the onset, maintenance, and treatment of several mental disorders, including symptoms of PTSD, depression, and anxiety disorder^24^. There is evidence that depressed individuals differ from controls regarding their ways of regulating their negative emotions in response to stressful events by using more frequently maladaptive emotion regulation strategies of rumination and catastrophizing, and using less frequently adaptive strategies of putting things into perspectives^25–27^. Additionally, maladaptive patterns of emotion regulation in anxious individuals can result in chronic avoidance and, thus as maintaining fear across time^28^.

One further mechanism by which exposure to early life stress may contribute to psychopathology is through a process of stress sensitization^29–32^, wherein individuals who have experienced early adversity have a lower threshold for developing psychopathological symptoms to recent stressors^33,34^. Consistent with the hypothesis, a prior study suggests that early life stress may sensitize limbic brain regions to adult trauma exposure in certain ways that further contribute to an enhanced vulnerability to mental illnesses^35^. Thus, it is important to explore the relationship between early adversity, recent stressors, and psychological symptom manifestation and severity.

The current study aimed at examining the relationship between early life stress, cognitive emotion regulation strategies, and mental disorders with high prevalence, namely PTSD, depressive and anxiety symptoms, among a sample of Syrian refugees. The ongoing Syrian Civil War has caused the largest refugee displacement crisis of our time. Since March 2011 approximately eight million people are internally displaced in Syria, and four million Syrians have been forced to flee to other countries seeking safety and protection^36–40^. Compulsory migration, the experience of traumatic events both within their country of origin and in the host countries as well as resettlement in unfamiliar cultural settings with challenging socio-economic circumstances generally leads to a higher risk for psychiatric morbidity, including symptoms of PTSD, depression, and generalised anxiety disorders^41–43^. However, studies evaluating psychiatric disorders among Syrian refugees show heterogeneity in prevalence rates of PTSD (from 20.5 to 35.7%), depression (from 20 to 43.9%) and anxiety disorder (from 19.3 to 31.8%), mainly due to different methodologies implemented^44–49^.

To the best of our knowledge, no research has investigated the interaction of early adversities, emotion regulation as a mediating factor, and mental health outcome among Syrian refugees. However, examining these relevant mental health factors in this sample group is crucial, since Syrian individuals may be less likely to seek specialty mental health treatment than other groups^50,51^ and at the same time, they are highly vulnerable to mental disorders due to multiple war exposure, flight and therewith associated traumatic experiences. Even when mental health and psychosocial services in the Syrian host communities are available, refugees may still be unable to access these services due to several factors, including cultural and linguistic barriers, the stigma associated with seeking mental health, and the power dynamics of the helping relationship^52^. Similarly, little attention is given to emotion regulation despite available evidence as a coping resource for positive changes and well-being^53,54^. Hence, we primarily sought to investigate whether emotion regulation plays a crucial role in regard to resilience to psychiatric symptoms among Syrian refugees.

In the present investigation, we sought to characterize the relationship between cognitive emotion regulation strategies, early life stress, PTSD, depressive, and anxiety symptoms. We hypothesised that participants with adverse childhood experiences would use more maladaptive and fewer adaptive cognitive emotion regulation strategies. Further, we hypothesised that these tendencies would influence current psychopathological symptoms. A further aim of the present study was to explore the potentially interactive role of recent trauma, namely exposure to the Syrian Civil War, in the stress sensitization effect of early adversity. According to the predictions of the stress sensitization model, it was hypothesised that individuals who have experienced greater extent to early adversity will demonstrate increased levels of symptom severity following subsequent trauma exposure in adulthood.

## Methods and materials

### Participants and procedures

During the 14-month study period from January 2017 to March 2018, eighty-nine Syrian refugees resettled in Germany (*n* = 49) and Jordan (*n* = 40) participated in the current research. In Berlin, participants were recruited at the Central Clearing Clinic, an outpatient institution by Charité—Universitätsmedizin Berlin, specialized in offering psychiatric services for refugees and collaborating with multiple refugee camps and civic initiatives. In Amman, participants were recruited by the German humanitarian NGO “Help-Hilfe zur Selbsthilfe”. Eligibility criteria included being 18–65 years of age, literate in Arabic language and having been exposed to the Syrian Civil War from 2011. Exclusion criteria included a lifetime diagnosis of psychotic disorder, bipolar disorder, personality disorder, mental retardation, any mental disorder due to a general medical condition and drug addiction. Participants were informed about the anonymity of information collected and their right to withdraw from the study at any time without giving a reason, or fearing of impacts on the services received by any governmental or nongovernmental organisation. Ethical approval (EA4/067/10) for the study was granted by the Institutional Review Board of Charité—Universitätsmedizin Berlin according to the Declaration of Helsinki. All subjects provided written informed consent and were financially reimbursed for participation.

### Questionnaires

The severity of depressive symptoms was assessed using the total score of the self-reported Patient Health Questionnaire-9 (PHQ-9)^55^. The PHQ-9 score can range from 0 to 27 since each of the nine items can be scored from 0 (not at all) to 3 (nearly every day) with higher scores indicating more severe depressive symptoms. The Arabic version of the PHQ-9 has been well validated^56–58^. In the present study, the PHQ-9 total score displayed good internal consistency (Cronbach’s *α* = .85).

The self-reported Generalized Anxiety Disorder-7 (GAD-7)^59^ measures the severity of anxiety symptoms with a range from 0 to 21. Each of the seven items can be scored from 0 (not at all) to 3 (nearly every day). The GAD-7 questionnaire was provided in a validated Arab version^57,60^, and was found to be highly reliable (Cronbach’s *α* = .86) in the current study.

The Harvard Trauma Questionnaire (HTQ)^61,62^ is a self-rated questionnaire assessing multiple refugee-specific facets of torture, trauma, and PTSD symptom severity that participants might have experienced in the home country, during the escape or in the host country. The first part comprises of 42 items illustrating traumatic events, such as lack of food and clean water, torture, rape, and murder of a family member or a friend, which are rated on a dichotomous scale: yes^44^ and no (0). The total score was the sum of all scores for each of the 42 items. The second part consists of an open-ended question asking the participants to describe the most hurtful/terrifying experience and to indicate whether this happened during the war, while fleeing, or in the host country. The third part encompasses 16 items, assessing PTSD symptom severity. Responses represent how often participants had experienced each trauma symptom (e.g., “feeling detached or withdrawn from people”, “difficulty concentrating”, or “trouble sleeping”). The HTQ total score is an average score, based on a range of responses from 1 (not at all) to 4 (extremely) for each symptom, with higher scores indicating an ascending level of PTSD symptom severity. It is a commonly used scale that has been validated in multiple cultures and languages^42,63^. For the current study, the Arabic version of the HTQ was used, which has already been validated with Iraqi refugees^63^. Previous studies have demonstrated sufficient validity and a good test–retest reliability^63,64^. In the current study, part one and three displayed good internal consistency with .89 and .87, respectively.

We used a retrospective self-report measure, the Childhood Trauma Questionnaire (CTQ)^65^ to assess the extent of early life stress that subjects had experienced. The CTQ consists of 28 items with five subscales. Items are rated on a five-point frequency scale from 1 = never true to 5 = very often true and summed up to give a total score for each type of trauma, ranging from 5 to 25 with higher scores indicating a more extensive exposure to that kind of stressful experience. Previous studies have demonstrated good convergent and discriminant validity, as well as good sensitivity and at least satisfactory specificity for the CTQ total score^66,67^. In the present study, the internal consistency of the CTQ total score is satisfying, with Cronbach’s *α* = .84.

The Cognitive Emotion Regulation Questionnaire (CERQ)^17^ was used to evaluate cognitive emotion regulation strategies used to respond to stressful events. It is a 36-item inventory that uses a five-point Likert scale to assess nine strategy subscales. In the present study, the nine subscales were categorized into maladaptive (CERQ- M) and adaptive (CERQ-A) strategies, and scores for both were summed from the relevant subscales. The Arabic version of the CERQ shows solid convergent validity and moderate to high reliabilities for each subscale^68^.

### Statistical analysis

Covariate distribution was investigated with *t*-tests, contingency tables, and Pearson or Spearman correlation for normally and non-normally distributed variables, respectively. Descriptive statistics are reported as mean +/− standard deviation. We planned a simple mediation analysis with bootstrapping techniques using the PROCESS macro for SPSS (version 3.0; Hayes, 2015). Overall, we performed six models, using consistently the total CTQ score (early life stress) as an independent variable. HTQ (PTSD), PHQ-9 (depression), and GAD-7 (anxiety) scores served as dependent variables in separately calculated models. CERQ-M (maladaptive cognitive emotion regulation) and CERQ-A (adaptive cognitive emotion regulation) subscores from the CERQ were mediating variables each time while controlling for age, gender, and educational level. We performed 10,000 bootstrap samples to generate a 90% bias-corrected confidence interval of the indirect effect *a* × *b*. In our mediation analysis, the *a* path represented the path from early life stress to adaptive/maladaptive cognitive emotion regulation, and the *b* path represented the impact of the mediator, adaptive/maladaptive cognitive emotion regulation, on PTSD, depressive, and anxiety symptoms. The output from our model also included path *c*, the total impact of early life stress on adulthood PTDS/depressive/anxiety symptoms, and *c*′, the direct impact of early life stress on PTDS/depressive/anxiety symptoms when accounting for adaptive/maladaptive cognitive emotion regulation. The Sobel-test was also used to confirm the significance of our mediation effects. The significance threshold was set at *p* < 0.05 and a one-tailed test was chosen for hypothesis testing. Additionally, we conducted a moderation model with total CTQ score serving as an independent variable, while HTQ, PHQ-9, and GAD-7 total scores were used again as dependent variables in separately calculated models. In this model, the first part of HTQ, representing adult trauma, was the moderating variable. To adjust for possible confounding effects, we included gender, age, and educational level as covariates. All statistical analyses were carried out using Predictive Analysis Software, version 25.0 (SPSS Inc., 2017).

## Results

### Demographics and psychiatric symptoms

Table 1 presents the demographic characteristics and social circumstances of the eighty-nine Syrian refugee participants. The mean age was 34.0 (±10.18) years, and 53.4% (*n* = 47) of the participants were female. Of the total sample, 59.1% were married, 36.4% were single, 1.1% were widowed, and 3.4% were divorced. 19.3% have a master’s degree, 13.6% a bachelor’s degree, 27.3% a high school certification, and 39.8% a lower or none school graduation. During the journey to the host country (*n* = 49 in Germany and *n* = 40 in Jordan), 67.0% fled with family members, 5.7 % with friends or acquaintances, while 27.3% crossed the borders alone. The mean time since the flight from Syria was 43 (±20.79) months, and the mean time of the resettlement in the host country was 39 (±21.6) months.

**Table 1:** Demographic characteristics of participants.

Characteristic	Mean (SD, range)/%
Age	34.0 (10.18, 41)
Gender (Female)	53.4%
Marital status (Married)	59.1%
Educational level (High school)	27.3%
Escape with family	67.0%
Months escaped from Syria	43 (20.79, 80)
Months resided in host country	39 (21.6, 84)

Table 2 summarizes all clinical outcomes, including mean, standard deviation, and range. The overall mean PHQ-9 score was 10.22 (±5.81), which is below the level for clinically significant depression. In this sample, 21.3% of participants met the cut-offs for mild, 29.2% for moderate, 30.3% for moderately severe, and 18% for severe depression. The mean GAD-7 score was 8.75 (±5.02), indicating, on average, mild anxiety in the study sample. 27% of respondents were above the cut-offs for mild, 36% for moderate, and 34.8% for severe generalized anxiety disorder. Using the HTQ, 30.3% of refugees met the cut-off for PTSD. The mean CTQ score was 35.31 (±9.76), indicating that participants reported moderate to severe childhood traumatic experiences. 29.5% of respondents reported no history of early life stress, 6.8% reported mild, 22.7% moderate, and 40.9% severe level of early life stress. 26.1% (±2.9) of subjects indicated having experienced emotional abuse, another 20.5% (±2.74) reported physical abuse, and 29.5% (±2.15) sexual abuse. 36.4% (±3.33) of participants reported emotional neglect, and 34.1% (±2.45) experienced physical neglect. Mean CERQ-M and CERQ-A scores were 45.49 (±10.0) and 69.2 (±12.28), respectively. The mean CERQ-M score (46.33 ± 11.20) was similar, and the mean CERQ-A score (56.41 ± 14.01) was higher than those of Korean patients in a comparable study^69^. An independent-samples *t*-test was conducted to compare the sociodemographic variables of the two subsample groups: Berlin and Amman. There was a significant difference in mean age between refugees in Berlin (*M* = 30.13, SD = 8.03) and Amman (*M* = 38.65, SD = 10.62); *t*(86) = −4.28, *p* < .001. The mean CERQ-M score for refugees in Amman (*M* = 48.91, SD = 9.33) was significantly higher than that of refugees in Berlin (*M* = 42.58, SD = 9.71); *t*(85) = −3.09, *p* = .003. Concerning clinical symptoms in both cohorts, results for depressive (8.31 in Berlin and 9.55 in Amman, PHQ-9 score) and anxiety symptoms (7.89 in Berlin and 9.60 in Amman, GAD-7 score) were comparable and therefore both at the cut-off threshold from mild to moderate symptom severity. Furthermore, a similar amount of trauma experiences was marked in both groups of refugees (16 items in Berlin, 18 items in Amman out of 43 items of the first part of HTQ). With a cut-off score for current PTSD set at >2.5, participants from Berlin presented PTSD symptoms bordering the diagnostic threshold (2.11). Similar to the Berlin cohort, participants from Amman displayed post-traumatic stress symptoms at the diagnostic boarder (2.31). Interestingly, statistical comparisons regarding clinical outcomes between subsamples demonstrated significant differences in PTSD symptoms (*p* < .04). We found substantial comorbidity among those refugees with psychological symptoms: while 12 (=13.5%) of the 89 respondents were suffering from one disorder only, 40 (44.9%) were screened positively for two and 24 (27%) for all three diseases. Remarkably, all refugees with PTSD symptoms in our sample were suffering simultaneously from depressive and anxiety disorder (*n* = 24).

**Table 2:** Clinical characteristics of participants.

Characteristic	Mean (SD, range)
Depression (PHQ-9)	10.22 (5.81, 26)
Anxiety (GAD-7)	8.75 (5.02, 20)
Posttraumatic disorder (HTQ)	2.2 (0.53, 2.44)
Early life stress (CTQ)	35.31 (9.76, 38)
Maladaptive cognitive emotion regulation strategies (CERQ-M)	45.49 (10, 47)
Adaptive cognitive emotion regulation strategies (CERQ-A)	69.2 (12.28, 58)

### Correlation for early life stress, maladaptive/adaptive cognitive emotion regulation strategies, current posttraumatic disorder, depression, and anxiety

The correlation matrix for all variables is provided in Table 3. Early life stress was positively correlated with the use of maladaptive cognitive emotion regulation strategies (*r* = .181, *p* < .05), with PTSD (*r* = .291, *p* < .01), depression (*r* = .351, *p* < .01), and anxiety (*r* = .287, *p* < .01). Maladaptive cognitive emotion regulation strategies were positively correlated with PTSD (*r* = .506, *p* < .01), anxiety severity (*r* = .374, *p* < .01), and depressive symptoms (*r* = .344, *p* < .01). Early life stress, PTSD, depressive, and anxiety symptoms were not significantly correlated with adaptive cognitive emotion regulation strategies.

**Table 3:** Correlation among early life stress, cognitive emotion regulation strategies, post-traumatic disorder, depression, and anxiety.

	CTQ	CERQ-M	CERQ-A	PTSD	PHQ-9	GAD-7
CTQ						
CERQ-M	.181*					
CERQ-A	.076	.111				
PTSD	.291**	.506**	−.161			
PHQ-9	.351**	.344**	−.107	.711**		
GAD-7	.287**	.374**	−.09	.708**	.768**	

*CTQ* childhood trauma questionnaire (early life stress), *CERQ-M* maladaptive subscales of cognitive emotion regulation questionnaire (Maladaptive cognitive emotion regulation), *CERQ-A* adaptive subscales of cognitive emotion regulation questionnaire (Adaptive cognitive emotion regulation), *PTSD* posttraumatic stress disorder, *PHQ-9* patient health questionnaire-9 (Depression), *GAD-7* generalized anxiety disorder-7(Anxiety),

*p < .05.

**p < .01.

### Model of early life stress and PTSD/depression and anxiety severity mediated by cognitive emotion regulation strategies

**Fig. 1:**
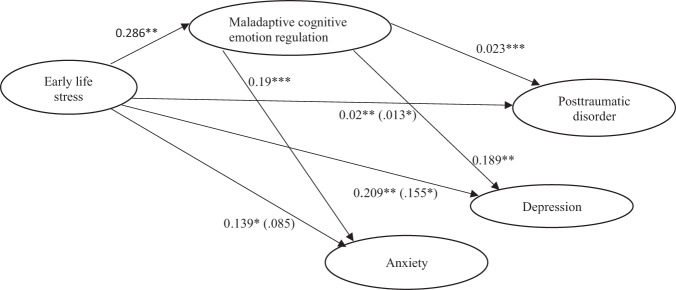
Meditation relationship. Mediation of relationship between early life stress and current posttraumatic disorder, depression and anxiety through maladaptive cognitive emotion regulation strategies. Note, *p < .05, **p < .01, ***p < .001.

Figure 1 displays the relationship between early life stress and later posttraumatic disorder, depressive, and anxiety symptoms as mediated by maladaptive cognitive emotion regulation strategies. The total effect of early life stress on current posttraumatic disorder is estimated as *c* = .02 with *p* < .01, and the direct effect is estimated as *c*′ = .013 with *p* < .05. The path coefficients were both significant: the path from early life stress to maladaptive cognitive emotion regulation strategies (*ß* = .286, *p* < .01) and the path from maladaptive cognitive emotion regulation strategies to PTSD (*ß* = .023, *p* < .001). The bootstrapping index for an indirect effect (*a* × *b* = .007) was significant when maladaptive cognitive emotion regulation strategies were included as mediating variables since the 90% confidence interval does not include zero [.003, .011]. Therefore, the mediating effect of overall maladaptive cognitive emotion regulation strategies on the relationship between early life stress and PTSD was significant.

There were significant indirect effects (*a* × *b* = .054) of early life stress on adulthood depressive symptoms through maladaptive emotion regulation strategies with a 90% confidence interval that does not include zero [.015, .108]. The direct path from early life stress to depression was also significant (*c*′ = .155, *p* < .05), showing that maladaptive cognitive emotion regulation strategies partially mediated the association between early life stress and later depression severity.

The total effect of early life stress on anxiety is estimated as *c* = .139 with *p* < .05 and the direct effect is estimated as *c*′ = .085 with *p* = .164, thus not significant. However, the path coefficients from early life stress to maladaptive cognitive emotion regulation strategies (*ß* = .286, *p* < .01) and from maladaptive cognitive emotion regulation strategies to anxiety (*ß* = .19, *p* < .001) were both significant. The bootstrapping index for an indirect effect (*a* × *b* = .054) was significant when maladaptive cognitive emotion regulation strategies were included as mediating variables because the 90% confidence interval does not include zero [.017, .105], suggesting that maladaptive cognitive emotion regulation strategies fully mediated the relationship between early life stress and current anxiety symptoms.

Neither the paths from early life stress to adaptive cognitive emotion regulation strategies nor from adaptive cognitive emotion regulation strategies to PTSD, depression, and anxiety were significant (all *p* > .05). The bootstrapping index for an indirect effect (*a* × *b*) was not significant when adaptive cognitive emotion regulation strategies were included as mediating variables. Therefore, the mediating effect of overall adaptive cognitive emotion regulation strategies on the relationship between early life stress and psychiatric symptoms was not significant.

### Model of early life stress and PTSD/depression and anxiety severity moderated by adult trauma

To examine whether experiences of adult trauma have a moderating effect on the relationship between early life stress and psychopathology, we applied a moderation model. Regarding our sample, there was no significant moderating influence of current trauma on the relationship between early life stress and mental illnesses (all *p* > .05).

## Discussion

In the current investigation, a mediation model was tested in order to examine the relationship between early life stress, current PTSD, depressive, and anxiety symptoms, as well as cognitive emotion regulation strategies among a Syrian refugee sample. Consistent with our hypothesis, maladaptive strategies partially mediated the effect of early life stress on PTSD and depressive symptoms of Syrian refugees. Our results are congruent with prior research proposing that the use of maladaptive strategies is an important mechanism underlying the negative effect of early life stress on psychological dysfunctions and early traumatic experience, which can further lead to impaired emotion regulation in later life^70^. Maladaptive appraisal originating from early life stress might, in turn, cause vulnerability to various mental health symptoms^71,72^.

One recent study with a clinical sample provided support for the mediating role of emotion regulation in the association between early life stress to both depression severity as well as lifetime persistence^73^. Within this sample, bootstrapping-enhanced mediation analyses indicated that specific emotion regulation skills significantly mediated the relationship between early life stress and depression severity. Another study examined the mediating role between emotion regulation strategies, current depression, and comorbid anxiety with respect to specific types of trauma^69^. Accordingly, emotional neglect was associated with difficulties in adaptive emotion regulation, whereas the mediation effect of maladaptive strategies was restricted to emotional abuse.

Notably, in our mediation model, maladaptive emotion regulation fully mediated the relationship between early life stress and anxiety symptoms, which is in line with findings of a study with a Korean cohort^69^. Consequently, there is an enormous need for continuous research investigating whether different psychopathologies are related to the use of specific emotion regulation strategies. A recent study suggested that the usage of maladaptive strategies can be considered as a general feature of depression and anxiety disorders^74^. However, anxious individuals attempted to suppress their emotions more likely than their depressed counterparts, whereas patients with depressive symptoms reported having used rumination more frequently than their anxious counterparts^74^. It will particularly be relevant for future research to identify, which concrete strategies are more protective or risk factors for certain types of psychiatric symptoms. Thus, subsequently, it can be ensured that these strategies are specifically targeted by prevention and intervention programmes.

However, in contrast to our assumptions, the mediating effect of adaptive coping strategies on the relationship between early life stress and psychiatric symptoms showed no significant associations. Interestingly, this result indicates similarity to a prior study, claiming that the indirect effect of adaptive emotion regulation strategies was weaker compared to maladaptive emotion regulation strategies^69^. Another study found out that maladaptive strategies (i.e., rumination, suppression, and avoidance) were more strongly associated with psychopathology than adaptive strategies (i.e., reappraisal, acceptance, and problem-solving), providing the support that adaptive strategies might play a minor role in the cognitive emotion regulation process compared to maladaptive strategies^75^. One possible explanation is that in contrast to maladaptive strategies, the implementation and following effects of adaptive strategies might depend on the context. For example, reappraisal might only be used adaptively if at all possible, whereas rumination seems to be maladaptive across time^76^.

Additionally, the effects of cognitive processes may depend on the clinical symptom severity^77^. For instance, individuals with mental disorders may fail to respond to stress with reappraisal because their maladaptive tendencies may be distinct to a significantly greater degree than their adaptive abilities. Thus, displaying a weaker significant indirect effect of adaptive strategies on the relationship between early life stress and adult symptom severity^75^.

Furthermore, we considered that adaptive coping strategies might be a potential moderator between early life stress and mental health problems, instead of having a mediating role. In a recent study, a significant interaction between the habitual usage of reappraisal (thought to be adaptive) and the exposure of emotional abuse on neural networks was revealed^78^. Based on this study, we tested a moderation model with early life stress and PTSD/depressive/anxiety symptoms as well as an adaptive emotion regulation strategy as moderating variable, while controlling for sociodemographic variables such as age, gender, and educational level. However, in our moderation model, there was no significant interaction between adaptive strategies and adverse childhood experiences. Thus, based on our data, we were not able to confirm the role of adaptive coping as a moderator between early life stress and adult psychopathology. More research is required in this field in order to further investigate the exact role of adaptive emotion regulation regarding the relationship between early life stress and psychiatric symptoms.

Regarding our sample, there was no significant interaction of war exposure on the relationship between early life stress and psychopathology. Based on our current cross-sectional data with refugees, it seems that early life stress and later life trauma seem to be independently associated with mental disorders. A recent study suggests similar outcomes in a military veteran sample showing that early life stress, combat exposure, and adult PTSD differentially predict alterations in amygdala and hippocampus connectivity^79^. However, it remains unclear whether war exposure is associated with stress sensitization and, if so, whether this effect is lasting or temporary. Future research is required to identify through which mechanisms the exposure to stress in early and later life lead to current mental problems. Therefore, it is essential to further investigate the occurrence and duration of stress sensitization prospectively following exposure to current stressors using longitudinal study designs.

Several limitations need to be considered in this study. First, all variables were assessed with self-report questionnaires. Generally, retrospective assessments rely on the accuracy of the participant’s memory and some types of mental disorders are associated with certain memory distortions. For example, avoidance and gaps in memories concerning traumatic events are major symptoms of PTSD^21^, and are also related to symptoms of depression and anxiety^75^. Retrospective reports of particularly early life stress may be prone to reporting bias. Notably, in our sample the prevalence of early life stress, especially for severe level of adverse childhood experiences, was high. Memory recalls of early adversity have been questioned for their accuracy as they may be influenced by current psychopathology^21,75^. Indeed, in the present study clinically significant symptoms of PTSD, depression, and anxiety were illustrated among Syrian refugees. However, previous research identified several predictors for robust memory, e.g., older age when the abuse ended, more severe experiences, and reported high levels of PTSD symptom severity^80,81^. Therefore, there is little reason to link psychopathology with less reliable and valid reports of early adversity^82,83^. Nevertheless, additional research is needed to explore the processes through which early life stress contributes to enhanced memory. Additionally, recent findings suggested that retrospective and prospective measures of early life stress may identify different groups of individuals, and therefore need to be considered separately^84^. Thus, assuming that the associated health outcomes and underlying risk mechanisms are the same in both groups may be inaccurate^84^. Second, the mediation model is cross-sectional, which limits any firm conclusions regarding the causality or temporal onset of emotional dysregulation and psychopathological symptoms^85^. Prospective longitudinal studies serially assessing changes in emotion regulation ability and mental health outcomes are required. Third, other potentially impactful factors on the current symptomatology (i.e., onset or length of the trauma and relationship with the perpetrator) were not evaluated with respect to early life stress. Fourth, cognitive emotion regulation is only a limited part of emotion regulation. Other types of maladaptive appraisals such as avoidance or suppression play an important role in the psychopathology of PTSD, depression, and anxiety^75^ and, therefore might be relevant regarding the relationship between early life stress and the aforementioned mental disorders. Fifth, we recruited Syrian refugees resettled in Amman as well as in Berlin. Thus, differences in the duration of the flight, cultural, and language challenges, and socioeconomic circumstances might also influence current symptom severity that needs to be evaluated in further investigations by our research group. As differences regarding PTSD symptom severity between subsamples were significant (*p* < .04), we calculated our mediation model for both groups separately. For the cohort in Berlin, we could confirm the significant mediating effect of maladaptive cognitive emotion regulation strategies on the relationship between early life stress and PTSD symptoms (*p* < .01). Yet, related to the cohort in Amman, the mediating effect of maladaptive cognitive emotion regulation on the relationship between early life stress and PTSD symptoms was not significant (*p* > .05). Thus, there is a need to explore possible reasons why Syrian refugees resettled in Amman suffer more frequently from severe PTSD symptoms than those resided in Berlin and, instead of maladaptive appraisal, which potential mediators may influence the link between early adversity and psychopathology among Syrians seeking refuge in Amman.

Despite its limitations, the present study provides evidence for the mediating role of maladaptive cognitive emotion regulation between early life stress and current PTSD, depressive, and anxiety symptoms in a Syrian refugee population. This is of relevance since the migration of vulnerable groups is a global challenge of increasing importance. Cognitive emotion dysregulation may be an important factor for patients who experienced early life stress and currently present with PTSD, depressive, and anxiety symptoms. Consequently, developing therapies that target emotion dysregulation can help in further enhancing the effectiveness of current treatments and prevention strategies and thus strengthen the resilience of Syrian individuals to mental health problems.
